# Probing the Electronic and Opto-Electronic Properties of Multilayer MoS_2_ Field-Effect Transistors at Low Temperatures

**DOI:** 10.3390/nano13162333

**Published:** 2023-08-14

**Authors:** Sujoy Ghosh, Jie Zhang, Milinda Wasala, Prasanna Patil, Nihar Pradhan, Saikat Talapatra

**Affiliations:** 1 School of Physics and Applied Physics, Southern Illinois University, Carbondale, IL 62901, USA; sujoy.kittu@siu.edu (S.G.); milinda.wasala@siu.edu (M.W.); prasanna@siu.edu (P.P.); 2 Center for Nanophase Materials Sciences, Oak Ridge National Laboratory, Oak Ridge, TN 37830, USA; 3 Department of Chemistry, Physics and Atmospheric Science, Jackson State University, Jackson, MS 39217, USA; nihar.r.pradhan@jsums.edu

**Keywords:** 2D materials, field effect transistors, opto-electronic transport, photodetector

## Abstract

Transition metal dichalcogenides (TMDs)-based field-effect transistors (FETs) are being investigated vigorously for their promising applications in optoelectronics. Despite the high optical response reported in the literature, most of them are studied at room temperature. To extend the application of these materials in a photodetector, particularly at a low temperature, detailed understanding of the photo response behavior of these materials at low temperatures is crucial. Here we present a systematic investigation of temperature-dependent electronic and optoelectronic properties of few-layers MoS_2_ FETs, synthesized using the mechanical exfoliation of bulk MoS_2_ crystal, on the Si/SiO_2_ substrate. Our MoS_2_ FET show a room-temperature field-effect mobility μ_FE_ ~40 cm^2^·V^−1^·s^−1^, which increases with decreasing temperature, stabilizing at 80 cm^2^·V^−1^·s^−1^ below 100 K. The temperature-dependent (50 K < T < 300 K) photoconductivity measurements were investigated using a continuous laser source λ = 658 nm (E = 1.88 eV) over a broad range of effective illuminating laser intensity, P_eff_ (0.02 μW < P_eff_ < 0.6 μW). Photoconductivity measurements indicate a fractional power dependence of the steady-state photocurrent. The room-temperature photoresponsivity (R) obtained in these samples was found to be ~2 AW^−1^, and it increases as a function of decreasing temperature, reaching a maximum at T = 75 K. The optoelectronic properties of MoS_2_ at a low temperature give an insight into photocurrent generation mechanisms, which will help in altering/improving the performance of TMD-based devices for various applications.

## 1. Introduction

Owing to the success of synthesis and applications of graphene and graphene-based materials, the scientific community has started to explore the possibility of isolating 2D atomic layers from other layered materials to investigate fundamental physics in non-carbon-based 2D crystals as well as for developing them in future electronic and opto-electronic applications. Recent advances in this area have shown that several other materials, such as hexagonal boron nitride (h-BN), transition metal di-chalcogenides (TMDCs), including MoS_2_ and WS_2_, and group III-VI layered semiconductors (e.g., GaSe, InSe, etc.), can be easily exfoliated from bulk crystals or grown using the CVD technique from a single to a few atomic layers [1,2,3,4]. In addition to the 2D structure, these materials also cover a wide range of the spectrum, from the insulator to semiconductors as far as the electrical properties are concerned [4,5,6,7]. For example, hexagonal B-N has a layered structure very similar to graphene, but with a very large band gap [5], whereas TMDs MX_2_ (M=Mo, W, Re, etc.) have band gap ranges from 0.8 eV to 3 eV and tunable as a function of the number of layers [6]. Some of these crystals, such as MoS_2_, MoSe_2_, WS_2_, WSe_2_, etc., show indirect to direct band gap transition when they swift from a bulk to single-atomic-layer structure due to the quantum confinement effect [7]. On the other hand, InSe [8,9,10], ReS_2_ [11], ReSe_2_ [12] etc., show direct band gap independent to the number of layers. The magnitude of the band gap weakly depends upon the number of layers for ReS_2_, where single-layer ReS_2_ shows a gap at 1.5 eV and 1.58 eV for monolayer [11]. Most of the TMDCs have moderate bandgap, which results in a higher ON/OFF current ratio exceeding 10^7^ and small subthreshold voltage swing ~70 mV/decade when exploited as FETs [13]. Apart from FET applications, semiconducting TMDCs also exhibit superior optoelectronic properties, which strongly depend on the number of layers [14]. Additionally, some TMDCs are also strongly correlated electronic materials, exhibiting properties like metal-insulator quantum phase transition, superconductivity, Mott insulator, etc. [15,16,17].

Among the several abovementioned TMDs, the most extensively studied TMD material is MoS_2,_ with thickness ranging from monolayer to several tens of layers [18]. Monolayer MoS_2_-based FET deices show electronic mobility 200 cm^2^·V^−1^·s^−1^ along with a very high ON/OFF ratio of 10^8^ [13]. Apart from FET applications, numerous studies show strong photo-response properties of 2D-MoS_2_-based photodetectors with high photo-responsivity and detectivity [19,20]. However, performances of thin MoS_2_-based photodetectors are mainly limited by low light absorption (5–10%) [20]. Further, most of the past studies are primarily focused on the room temperature as well as low-temperature electronic transport properties of either monolayer or bilayer MoS_2_-based devices. We have shown in our previous study using indium-selenide-based FET [9] that both the transport mechanism as well as key figures of the merits of an FET device fabricated using a 2D-layered material depends on the number of layers. Additionally, other factors such as surface-induced trap charges, defects inside the MoS_2_ channel, etc., also result in detrimental photo-response properties, such as short carrier lifetime and persistent photoconductance [21]. In comparison, multilayer MoS_2_-based devices have several advantageous features, e.g., high density of states resulting in a high drive current as well as tunable electrical properties (n-type and p-type) [22]. However, multilayer MoS_2_-based FETs and corresponding photo-detection properties have not been extensively studied. In this work, we have fabricated FET devices based on several-layers-thick MoS_2_ and have systematically studied their electronic and photo-response properties. Multilayer MoS_2_ FETs show n-type behavior with room-temperature electronic mobility ~40 cm^2^·V^−1^·s^−1^. The electronic mobility increases by almost double ~80 cm^2^·V^−1^·s^−1^ at low temperatures. We further found that maximum photo-responsivity reaches up to ~10 A/W at low temperatures while operating the FETs under the ON condition. 

## 2. Synthesis and Device Fabrication

A few layers of MoS_2_ were obtained using a mechanical exfoliation technique from commercially available bulk MoS_2_ crystal (SPI Supplies). The exfoliated flakes/thin layers crystals were subsequently transferred on to a chosen substrate for device fabrication. For device fabrication, we chose a suitable MoS_2_ flake. Using an optical microscope, a TEM square mesh grid (Electron Microscopy Sciences, Hatfield, PA, USA) was placed carefully on the top of the flake. This mesh grid works as a shadow mask for thermal deposition to put in direct electrical contacts. In this method, we do not use any photoresist or polymers and avoid further fabrication-process-related impurities which may lead to detrimental device performances [23]. After placing this shadow mask, the system was then mounted inside the thermal evaporator. We deposited Cr (10 nm)/Au (100 nm) for the contacts at the chamber pressure 10^−6^ Torr. The height of the MoS_2_ flake was measured using atomic force microscopy, ~9–10 nm thick, which corresponds to ~13–15 layers of MoS_2_. After deposition of the metal electrodes, the devices were annealed at a high temperature in an inert atmosphere.

## 3. Results and Discussion

**Figure 1:**
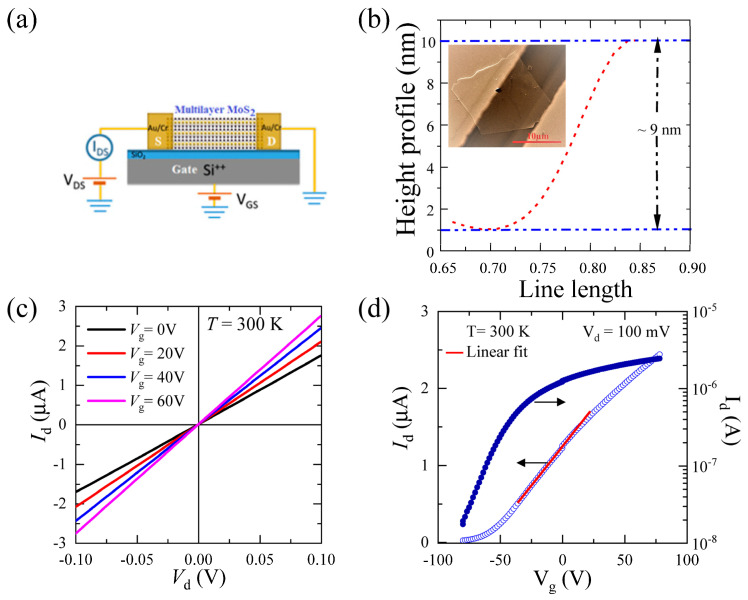
(**a**) Schematic of the three-terminal device architecture with laser light illumination in the channel region. (**b**) Height profile of the MoS_2_ flakes (red dashed line) extracted from the optical image of the device (inset); (**c**) I_d_-V_d_ characteristics of the MoS_2_ FET device under different back-gate voltages. (**d**) Transfer (I_d_-V_g_) characteristics of the MoS_2_ FET in linear scale (left axis) and log scale (right axis). The red color line represents the liner fit.

Three terminal field-effect transistor (FET) measurements were performed on the as prepared MoS_2_ devices (described in the previous section) under high vacuum ~10^−6^ Torr and at different sample temperatures 20 K ≤ T ≤ 280 K. To achieve the FET operation, a small d.c voltage (V_d_) of 100 mV was applied between the source and drain contacts. The gate voltage was swept between −80 V and +80 V, and the corresponding drain-source currents (I_d_) were recorded. The fabricated devices were initially used to measure the electrical properties at room temperature. Figure 1a shows the schematic of the device with a laser light illuminating the whole device from the top. Figure 1b shows the optical micrograph image containing the 9–10 nm thick MoS_2_ device (inset). The channel length and width of the devices is ~10 µm and ~13 µm, respectively.

We used Keithley 2400 SMU to measure the drain-to-source current (I_d_) by biasing the drain-to-source voltage (V_d_), while gate voltages were applied using another Keithley 2410 SMU. The drain-to-source current (I_d_) as a function of the drain-to-source voltage (V_d_) at several gate voltages (V_g_) is shown in Figure 1c. The I_d_-V_d_ data look linear despite the Schottky barrier between the MoS_2_ and Cr metal interface due to higher thermionic emission processes at room temperature. The low-temperature I_d_-V_d_ data are presented later in this section, which reveal that the metal-semiconductor contacts are indeed of Schottky type. Figure 1d shows the FET transfer characteristics of our MoS_2_ device at 300 K temperature, showing I_d_ as a function of V_g_ at V_d_ = 100 mV. A typical n-type transistor operation was observed with a threshold gate voltage (V_th_) ~ −60 V. The drain-source current I_d_ reaches to the maximum saturation value of ~2 μA for gate voltages, V_g_ > 60 V and remains ~10 nA for V_g_ < −60 V. From this, we estimated the ON/OFF ratio to be ~10^2^. The low ON/OFF ratio can be attributed to the higher thickness of the MoS_2_ with a lower bandgap. The red line shows the linear fit of the I_d_-V_g_ characteristics plot and was used to extract the field-effect mobility of the device using the MOSFET transconductance formula given below,

(1) $$\mu =\frac{L}{w}\frac{1}{C}\left(\frac{dI_{d}}{dV_{g}}\right)\frac{1}{V_{d}}$$

where L is the channel length, W is channel width, and C is the capacitance per unit area of the gate dielectric.

This n-type FET operation has been previously observed in many mechanically exfoliated single-layer MoS_2_ devices. The field-effect electronic mobility was calculated from the linear region by using Equation (1) with an oxide layer thickness of 1000 nm. At 300 K, the field-effect mobility is ~46 cm^2^·V^−1^·s^−1^. This mobility value is similar to the previously obtained room-temperature mobilities of multilayered MoS_2_ devices [24] and higher than single-layer MoS_2_ devices [13,15]. However, with appropriate contact engineering, these mobility values can be further improved [25,26,27,28].

**Figure 2:**
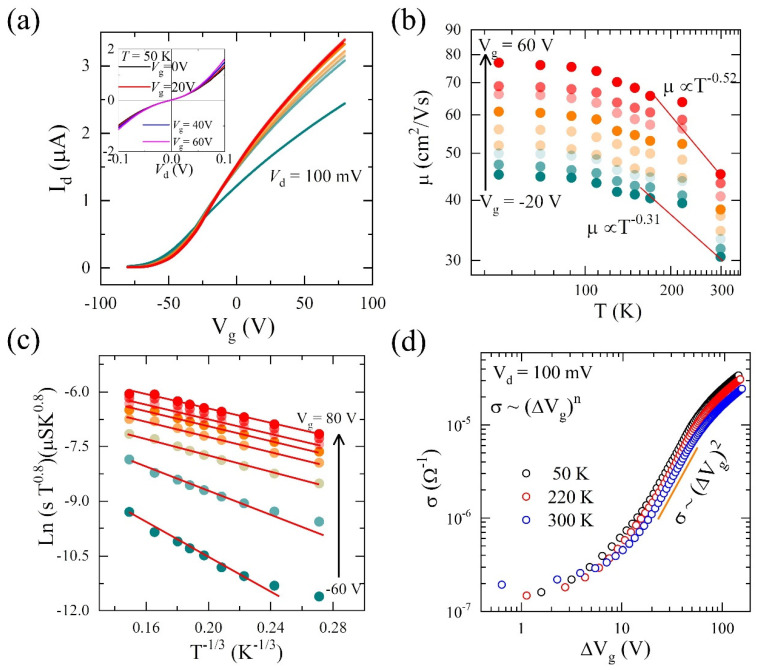
(**a**) I_d_-V_g_ characteristics of MoS_2_ FET at different temperatures (50 K ≤ T ≤ 300 K). (**b**) Temperature-dependent field-effect electronic mobility under different back-gate voltages. (**c**) Temperature dependence of conductivity (σ) and variable range hopping (VRH) at different back-gate voltages. (**d**) Variation in conductivity σ with ΔV_g_ at different temperatures.

This higher mobility is mainly due to the higher density of states and lower Schottky barrier/Ohmic contacts found in the case of our multilayer MoS_2_ device. Figure 2a shows the low-temperature FET transfer characteristics at different temperatures. We have calculated the back-gated field-effect mobilities at different temperatures, keeping the source-drain voltage constant (100 mV), as shown in Figure 2b. The mobility increases further up to ~80 cm^2^·V^−1^·s^−1^ as the temperature reaches 50 K, which is generally attributed to reduced phonon scattering [29].

The charge carrier transport in 2D semiconductors is mainly dependent on the carrier density or equivalently on the Fermi energy level E_F_ [30,31,32,33]. When the charge carrier density is “very-low”, the Fermi level E_F_ lies in the bandgap region without any mobile carriers. In this case, the system remains in a disorder-driven strongly localized insulating phase. In the case of an n-type semiconductor, the application of a positive gate-bias moves the Fermi level towards the conduction band edge. This results in the generation of mobile electrons by filling up the localized states by thermal excitation. Further increment in the gate bias shifts the Fermi level above the mobility edge, and thus the band transport becomes more dominant. Finally, when the carrier density becomes “very-high”, a strong apparent metallic phase is observed. At higher temperatures and at “very-high” doping level, the charge carrier transport is often influenced by the phonon effects and by short- and long-range scatterers, such as defects and charged impurities. The transition from the insulating state to the conducting state involves gradual progressive filling of the localized states or band edge disordered states arising from impurities and/or structural defects. At the intermediate carrier density level, charge carrier conduction occurs via the hopping of charge carriers through the localized states [34,35,36,37]. These type of conduction mechanisms can be described by the variable range hopping (VRH) model, which can be expressed by the following equation [34,38]:

(2) $$\sigma \;(n,\;T)=A.T^{m}\;\exp\;[{-(\frac{T_{0}}{T})}^{\gamma }]$$

where the exponent γ = 1/(d + 1), d is the dimension of the system (d = 1 for 1D system and d = 2 for 2D systems), m = 0.8 is an empirical constant, T is the temperature, T0 is the characteristic temperature, and σ (n, T) is the 2D conductivity.

To further confirm the charge transport mechanism process in our MoS_2_ devices, we plotted the natural logarithm of the conductance as a function of T^−1/3^ for different back-gate voltages, as shown in Figure 2c. Here we found that the charge transport for our MoS_2_ device follows 2D VRH mechanisms, as given by Equation (2), over a wide range of temperatures 50 K ≤ T ≤ 300 K. These 2D VRH mechanisms further support mobility T^−1/3^ dependence, similar to the results found in monolayer MoS_2_ devices [34].

In crystalline MoS_2_, the presence of a high density of localized states in the band-gap region leads to hopping transport when the Femi level moves through them upon changing the gate voltages. According to previous results [34], the physical origin of these localized states in MoS_2_ films is connected to the random potential fluctuations from the trapped charges at the MoS_2_-SiO_2_ interface. Since the screening of these trapped charges is relatively poor due to the large band gap of MoS_2_, a considerably long band tail thus exists from the interfacial traps which are randomly occupied during the processing of the device. For a 2D system with parabolic energy bands, if the main source of disorder arises from the randomly occupied interfacial traps, the scattering mechanisms are also expected to be dominated by charge impurity scattering [39]. This will further lead to:

(3a) $$\sigma \propto n\;\left(for\;screen\;coulomb\;impurity\right)$$

(3b) $$\sigma \propto n^{2}\;(for\;bare\;Coulomb\;impurity)$$

Therefore, to understand the scattering mechanism in our devices, we have plotted the conductance σ as a function of the change in the back-gate voltage (ΔV_g_) at different temperatures, as shown in Figure 2d. We found that for our MoS_2_ devices towards the higher gate voltages, σ almost follows the power law expression σ~ΔV_g_^2^ for all different temperatures, which indicates scattering from almost unscreened charge impurities in our MoS_2_ devices.

## 4. Optoelectronic Transport

**Figure 3:**
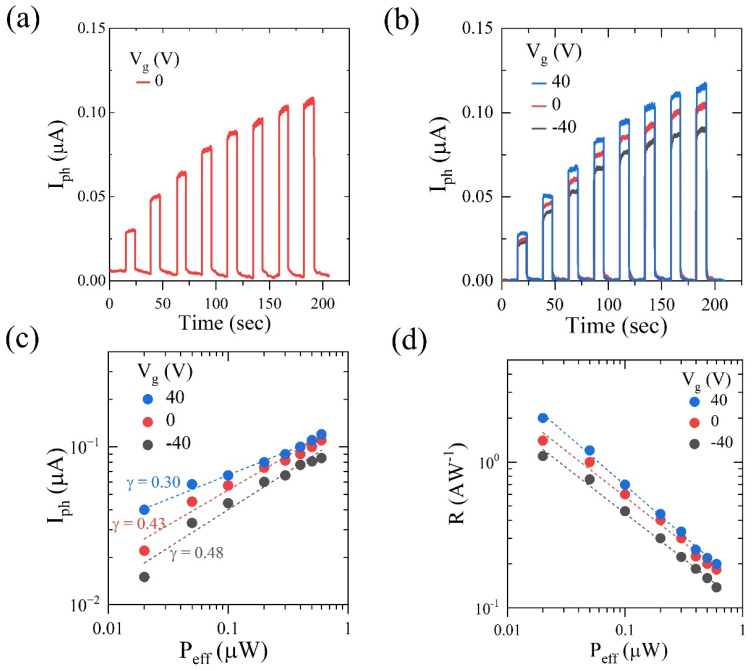
(**a**) Photocurrent vs. time at room temperature with no back-gate voltage and (**b**) with different back-gate voltages. (**c**) Photocurrent vs. effective power illumination in the whole channel region in log–log scale. (**d**) Responsivity vs. effective power under different back-gate voltages.

The optoelectronic properties of MoS_2_ FET were investigated by using a continuous wave laser with an illumination wavelength λ = 658 nm (E = 1.88 eV) and a spot size of ~3 mm in diameter. A larger laser spot size helps reduces photo-thermal effects, such as the photo-thermoelectric effect, photo-bolometric effect, etc., as both contacts are illuminated alike [40]. Additionally, laser illumination intensity (P_laser_) is scaled to an effective laser illumination intensity (P_eff_) owing to a larger laser spot size as P_eff_ = P_laser_ × A_device_/A_spot_, where A_device_ is the area of a device and A_spot_ is the area of a laser spot. Room-temperature optoelectronic transport measurements of an MoS_2_ FET are shown in Figure 3. A continuous laser was switched ON and OFF for an interval of ~10 s, and the corresponding drain currents were measured. With the data shown in Figure 3a,b, it can be estimated that the decay response times of this device studied are very fast, perhaps of the order of a few seconds; however, due to the limitation of the measurement system, an accurate determination of this parameter was not possible. Photocurrents (I_ph_) were estimated as a difference between the drain current under laser illumination (laser on) and the drain current under a dark current (laser off) as I_ph_ = I_d,laser ON_ − I_d,laser OFF_. The time-dependent response of the photocurrent at V_g_ = 0 V and various V_g_’s is shown in Figure 3a,b, respectively. It was observed that the photocurrent reverts to the drain current under dark conditions as soon as the laser is switched off, implying the presence of only a photoconductive/photogating effect. 

The photocurrent (I_ph_) extracted for three different applied gate voltages (V_g_ = −40 V, 0 V, 40 V) is plotted as the function Peff in log–log scale, as shown in Figure 3c. The photocurrent follows power law dependence on the effective laser illumination intensity as I_ph_ ∝ (P_eff_)^γ^. The value of the exponent is 0 ≤ γ ≤ 1, indicating the presence/absence of trap states in FET. In the absence of trap states, the exponent, γ = 1, and photocurrent generation mechanism follow a pure photoconductive effect. In the presence of trap states, the exponent becomes fractional (γ < 1) and the photocurrent mechanism manifests into a trap-dominated photoconductive effect, commonly known as the photogating effect. It should be noted that crossover of a photo generation mechanism from photoconductive to photogating because of an increased applied gate voltage has been observed in various 2D materials, such as In_2_Se_3_ [40], CuIn_7_Se_11_ [41], and ReSe_2_ [42], which indicates that trap states can be modulated. However, the fractional value of γ for all gate voltages (in Figure 3c) indicates that the trap states in MoS_2_ FET have a strong influence on the optoelectronic properties of MoS_2_ FETs.

The photo-responsivity (commonly known as responsivity) of a phototransistor can be estimated by R = I_ph_/P_eff_. The responsivity as a function of P_eff_ in log–log scale for three different applied gate voltages is shown in Figure 3d. In the case of a pure photoconduction-generated photocurrent (where photoconductive gain is absent), responsivity will have an upper limit, given by R = (η × e × λ)/(h × c) = η × λ/1240, where η is the quantum efficiency, e is the electron charge, h is the plank’s constant, and c is the speed of light [43,44]. For a laser with λ = 658 nm, a maximum R of 0.53 A W^−1^ can be achieved for η = 1 (100% conversion). For MoS_2_ FET, responsivity values greater than 0.52 (as seen in Figure 3d) indicate that the gain > 1. Gain can be estimated as τ_m_/τ_d_, where τ_m_ is the minority carrier lifetime and τ_d_ is the carrier drift or transit time [45]. For gain > 1, τm > τd, which means that minority carriers are immobile and trapped. This results in a trap-dominated photoconductive effect. As seen in Figure 3d, the responsivity decreases as the effective laser intensity increases, and this could be attributed to a decrease in the average carrier lifetime of minority charge carriers [43]. As light intensity increases, trap states gradually start filling up. At certain light intensities, all the trap states are filled, and a further increase in intensity will result in the generation of minority carriers that cannot be trapped. As a result of this, τ_m_ decreases, thus reducing the gain and the responsivity [43]. For MoS_2_ FET, a maximum R = 2 AW^−1^ at 300 K can be obtained at P_eff_ = 0.02 μW and V_g_ = 40 V. When the gain > 1, quantum efficiency (η) is known as the external quantum efficiency (EQE) and is defined as EQE = R (h × c)/(e × λ) = R × 1240/λ. We found a maximum EQE of ~380% at P_eff_ = 0.02 μW and V_g_ = 40 V.

**Figure 4:**
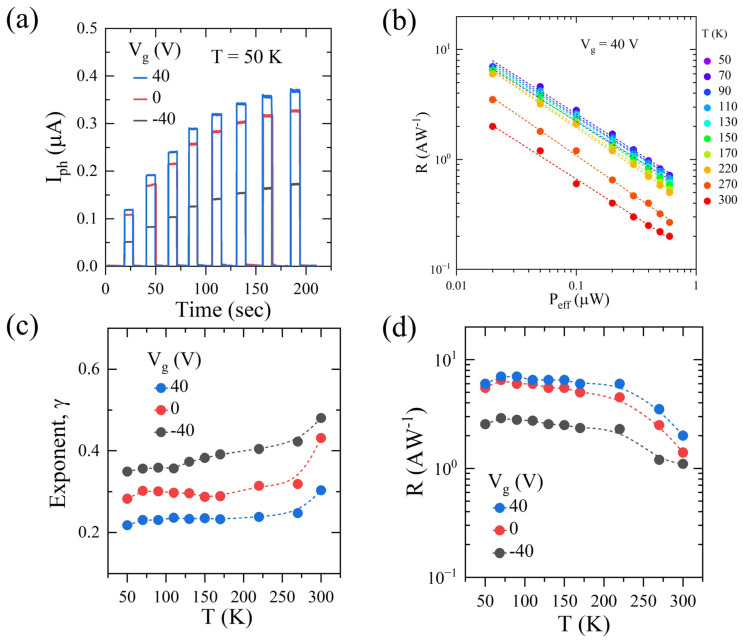
(**a**) Photocurrent vs. time under different back-gate voltages. (**b**) Responsivity variation with effective power and temperature under back-gate voltage of 40 V. (**c**) Exponent (γ) vs. temperature (extracted from (**c**)); (**d**) responsivity vs. temperature.

Temperature plays an important role in determining the strength of traps states. Thus, to emphasize the role of traps states on the photogeneration mechanism, we studied the optoelectronic properties at a low temperature (50 K < T < 300 K). Photocurrents at all studied temperatures show similar behaviors as that of those at room temperature. The time-dependent response of the photocurrent (at various V_g_’s) measured at the lowest temperature is shown in Figure 4a, and the photocurrent reverts to the drain current under the dark condition as soon as the laser is switched OFF. Responsivity is a function of P_eff_ at constant V_g_ = 40 V, and all the temperature studies are shown in Figure 4b. Responsivity increased as temperature decreased, indicating strong trapped states. 

The temperature dependence of γ and R is shown in Figure 4c,d, respectively. It is observed that as temperature decreases, gamma decreases, and, correspondingly, responsivity increases for all the studied gate voltages (V_g_’s). A strong dependence of γ and R have been observed in various 2D-materials-based FETs, and this was studied in detail in the case of ReSe_2_ [42]. A clear correlation between R and γ was observed, where lower γ corresponds to higher R. These parameters are determined by trap states, and these traps can be modulated by temperature and gate voltages. In a semiconductor where the steady-state Fermi level is away from the valence band, this results in mid-gap states being available for trapping. Lowering the temperature results in carrier freeze out and strongly trapped minority carriers, resulting in a strong photogating effect. Thus, responsivity increases at lower temperatures.

## 5. Conclusions

In conclusion, here we have presented the detailed temperature-dependent electronic and optoelectronic properties of few-layers MoS_2_ FETs. Our MoS_2_ FETs show mobility μ_FE_ ~40 cm^2^·V^−1^·s^−1^ at room temperature and 80 cm^2^·V^−1^·s^−1^ below 100 K. The temperature-dependent (50 K < T < 300 K) photoconductivity measurements show the room-temperature photoresponsivity (R) to be ~2 AW^−1^, and this increases as a function of decreasing temperature. Photoconductivity measurements indicate a fractional power dependence on the steady-state photocurrent, indicating trap-controlled optoelectronics properties. Understanding the optoelectronic properties of MoS_2_ at low temperatures will help in altering/improving the performance of TMD-based devices for various applications. Further, these studies may lead to the development of broadband photodetectors, as shown by Wei et al. [46], using ternary-selenide-based materials or components in neuromorphic computing [47].

## Data Availability

The data that support the findings of this study are available upon request to the authors.
